# Early Eating Patterns and Overweight and Obesity in a Sample of Preschool Children in South-East Poland

**DOI:** 10.3390/ijerph16173064

**Published:** 2019-08-23

**Authors:** Joanna Baran, Aneta Weres, Ewelina Czenczek-Lewandowska, Edyta Łuszczki, Grzegorz Sobek, Grzegorz Pitucha, Justyna Leszczak, Artur Mazur

**Affiliations:** 1 Medical Faculty, University of Rzeszów, al. Mjr. W. Kopisto 2a, 35-959 Rzeszów, Poland; 2 Faculty of Biology and Agriculture, University of Rzeszów, ul. Ćwiklińskiej 1, 35-601 Rzeszów, Poland

**Keywords:** BMI, children, obesity, overweight, preschool

## Abstract

The aim of this study was to assess the impact of a child’s diet in the first year of life (breastfeeding duration, introduction of solid meals to the diet, the time of starting nutrition consistent with an adult diet) on the prevalence of overweight and obesity in preschool age. Three-hundred children aged 4–6 were included in the analysis. The children’s height and body weight were assessed and their body mass category was determined based on the BMI (Body Mass Index) percentile. Parents provided a photocopy of the child’s health book (with information concerning breastfeeding period, start of eating the same meals as the rest of the family, etc.). Obese children were breastfed for the shortest time, cow’s milk was introduced to their diets the earliest, they started eating the same food as the rest of the family the earliest, and they received vegetables, fruits, cereals, and meat products in their diet the latest. The results of this study suggest that extending the breastfeeding period beyond 6 months, starting to feed the child the same meals as the rest of the family after 12 months of age, and later introduction of cow’s milk to the diet would reduce the risk of the occurrence of excessive body weight in preschool children.

## 1. Introduction

Childhood obesity is a significant public health problem around the world. Its prevalence is increasing year by year at a drastic pace. According to the WHO, from 1990 to 2016 there was an increase in the number of overweight and obese children under 5 years of age from 32 million to 41 million [1]. In South-Eastern Poland between 2008 and 2012, the prevalence of overweight in preschool children increased from 9.1% to 12.4% and obesity from 7.2% to 10.8% [2].

The main cause of overweight and obesity is the excessive consumption of calories, with little expenditure, and reduced physical activity. However, in the case of young children, whose bodies are only just developing, the way they are being fed and the provision of appropriate food products may be a cause. Authoritarian styles, rigidity, and restriction create a negative emotional context for eating which can be counter-productive to healthy self-regulation. Authoritative styles marked by high sensitivity, responsiveness, and expectations of self-control have been linked with a lower risk of obesity and flexible, but healthy, autonomous eating [3]. Processed, high-energy, low-health foods and sugar-sweetened drinks should be replaced with fresh products and water in both schools and family homes. Healthy eating habits should be cared for from infancy. This requires the parent to understand the relationship between diet and health, and to learn behavior that encourages and supports the development of healthy eating habits.

Numerous studies indicate the undoubted influence of length of breastfeeding on reducing the risk of overweight and obesity in children [4,5,6]. The mother’s milk contains the appropriate amount of nutrients necessary for the child, and no other milk can replace it [7]. Most people consume cow’s milk on a daily basis, and milk is also introduced to the diets of small children. Studies show diverging views on its effects on the prevalence of overweight and obesity in children [8,9,10].

The period when the diet of a small child is extended involves the introduction of vegetables, fruits, meat, cereals, etc. Fruits and vegetables are a source of, among others, potassium, folic acid, fiber, and vitamins A, C, and K. A diet rich in fruits and vegetables allows for the control of body weight and is associated with a reduced risk of chronic diseases in adulthood [11,12]. Studies show that eating fruit and vegetables in pregnancy and during infancy affects a child’s eating habits in later years [13,14,15].

The aim of this study was to assess the impact of a child’s diet in the first year of life (i.e., breastfeeding duration, introduction of solid meals to the diet, the time of starting nutrition consistent with an adult diet) on the prevalence of overweight and obesity in preschool age.

## 2. Materials and Methods

### 2.1. Data Collection

The study included 10 randomly selected preschool educational institutions from South-Eastern Poland. After obtaining the consent of the management of the facility, a meeting was held with the parents of the children and consent for each child’s participation in the study was given.

The parents who agreed for their child to be studied were required to complete a questionnaire describing the family (including questions about gender, age, child’s place of residence, parents’ BMI and level of education, etc.) and to provide a photocopy of the child’s health book (with information concerning birth weight, breastfeeding period, extension of the child’s diet, start of eating the same meals as the rest of the family, etc.).

Then, the children’s height and body weight were assessed:

- Body height measurement was taken using a seca 213 stadiometer. The subjects stood barefoot, with their back to the measuring part of the meter. Body height was measured three times, and the average value was taken in order to eliminate measurement error.
- Body weights were assessed using a Tanita BC-420MA analyzer. Subjects stood barefoot on the analyzer, and the upper limbs were placed along the torso, slightly away from it.

After collecting all the above data, each child’s current percentile BMI was determined with reference to Polish percentile grids, and their body mass category was determined based on the BMI percentile [16]. Current screening tests using anthropometric parameters of nutritional status recommend overweight and obesity based on centiles: overweight ≥85 centile, obesity ≥95 centile [17]. Analysis of population BMI distributions in the Polish OLA and OLAF studies, current representative samples for the national population of children and adolescents aged 3–18, shows that the 95th percentile of the BMI distribution in boys is significantly below the obesity limit, while the 85th percentile coincides with the overweight limit. For girls, the differences between the 85th and 95th percentiles and the overweight and obesity limits, respectively, are even greater. Adoption of the 95th percentile BMI distribution by age in Poland as limit values for the diagnosis of obesity—regardless of whether the WHO reference system or the current BMI distribution representative for the national population is used—will lead to over-recognition of obesity among children and adolescents, and adoption is suggested at the border of the 97th centile [16].

The children were also divided according to birth weight and duration of pregnancy into:

- Appropriate for gestational age (AGA)—birth weight appropriate for gestational age, between the 10th and 90th centile for gestational age;
- Small for gestational age (SGA)—birth weight below the 10th centile for gestational age;
- Large for gestational age (LGA)—birth weight above the 90th centile for gestational age [18].

### 2.2. Participants

The study involved 385 children attending kindergartens in South-Eastern Poland. A total of 324 children were admitted to the study after the parents’ written consent was obtained. After preliminary data analysis, it turned out that four people did not give a response regarding the time of introducing fruit and vegetables, ten people did not give an answer on the period of breastfeeding, seven did not give information about the beginning of eating the same food as the family, and three did not give information about the time when cow’s milk was introduced into the child’s diet. Finally, 300 children aged 4–6 were included in the analysis.

### 2.3. Statistical Analysis

Statistical analysis was carried out in the Statistica 13.1 (StatSoft, Inc., Tulsa, OK, USA). Non-parametric tests were used, such as the Spearman rank correlation test (used to assess the relationships between two numerical variables), and the ANOVA Kruskal–Wallis test (used to assess differences in results obtained in more than two groups). Calculations were also made of the odds ratio of the occurrence of a given phenomenon in the population (OR). The level of statistical significance was accepted as *p* < 0.05.

### 2.4. Ethical

The approval of the Bioethics Committee of the University of Rzeszów No. 18/12/2015 from 2 December 2015 was obtained for conducting the research.

## 3. Results

### 3.1. Characteristics of the Study Group

First of all, a characterization was made of the study group, which consisted of 162 girls and 138 boys. In this group, 12% of the children had excessive body mass, and among the parents of the children, nearly 71% of fathers and 32% of mothers were overweight or obese. In addition, the fathers were characterized in most cases by secondary education and mothers with higher education.

Taking into account the children’s parameters, it should be noted that 13.3% of the children were born too small in relation to gestational age, and that over half of the children (62.7%) were breastfed for less than 6 months. In addition, every fifth child started eating the same meals as the rest of the family before the age of 12 months. Detailed results are presented in Table 1.

### 3.2. The Impact of Diet in Early Childhood on Current Body Weight

Table 2 shows the average time at which the individual food products were introduced into the child’s diet as well as the duration of breastfeeding. Multivariate analysis did not show statistically significant differences between the time of introducing individual food products into the child’s diet and their current body weight category. However, it is worth noting that overweight and obese children were breastfed for the shortest time, cow’s milk was introduced to their diets the earliest, they started eating the same food as the rest of the family the earliest, and they received vegetables, fruits, cereals, and meat products in their diet the latest (Table 2).

On the other hand, the correlation between the current body mass of children and breastfeeding time was significant (*p* = 0.001). The children who were breastfed longer had a lower current body weight.

A statistically significant correlation was also found between the current baby BMI percentile and the time of introducing cow’s milk into their diet (*p* = 0.023). The children who were given cow’s milk later had a lower BMI percentile (Table 3).

An assessment was also made of which of the above factors had the largest impact on the current weight of the child. Although the influence of none of the variables were statistically significant, the period of breastfeeding was near the threshold of significance (*p* = 0.070). This factor most affected the child’s body weight in the future (Table 4); b * signifies how much the BMI would increase if the feeding time was extended by a month. Each month longer of breastfeeding represented a decrease in the BMI percentile by about 0.16 points.

The odds ratio of the occurrence of overweight and obesity in preschoolers was also checked in relation to breastfeeding and eating from the shared family table according to recommendations. There was a higher risk of obesity/overweight in the group of children who were breastfed for less time (<6 months) than in children who were breastfed for longer (>6 months)—OR = 1.81. In addition, children who started eating from the adult table after 12 months of age were less prone to overweight/obesity than children who started eating the same food as the family earlier (OR = 0.67) (Table 5).

## 4. Discussion

Overweight and obesity in children and adolescents constitute a modern-world problem that is very complex, starting from its causes, through diagnostics, to treatment and prevention. Various factors have been described in the literature that have a potential impact on the prevalence of overweight and obesity (including biological, social, cultural, socio-demographic, perinatal, and others). However, the primary factor that we can influence is child feeding.

Many researchers have analyzed the relationship between breastfeeding and body weight at a later time. WHO recommends that a child should be fed only breast milk for the first six months of his life [19]. The optimal practice in preventing childhood obesity is to directly support WHO recommendations for 6-month exclusive breastfeeding and prolonged breastfeeding in combination with solid food intake from 6 months of age [20]. Many studies have reported a low prevalence of breastfeeding in general or breastfeeding that exceeds 6 months [21,22]. In our study, we also observed that a smaller percentage of children were breastfed for more than 6 months. Breastfeeding seems to be protective against future weight gain in children. This is undoubtedly due to the delivery to the child of a natural product from the mother with the balanced composition that the child’s body needs. Human milk has nutritional, immunological, and trophic properties, hence its great importance for the growing and maturing body. Human milk contains—in concentrations and proportions optimal for the child—species-specific proteins, fats, carbohydrates, micro and macro-elements, and biologically active substances [23]. However, short breastfeeding, replaced with modified milk or other products enriched with sugars, promotes excessive weight gain in the child. Such results have been obtained in both non-Latino and Latino populations [24,25].

This is consistent with the results of our own research, where breastfeeding below 6 months nearly doubled the risk of overweight or obesity in preschool children. In addition, each month longer of breastfeeding had an influence on the lowering of the BMI percentile of current body weight, and the shortest period of breastfeeding was observed in obese children.

Discussions about the optimal time for introducing complementary foods have focused mainly on the impact that this would have on the later risk of allergies. Initially, it was thought that delaying the introduction of certain types of food could reduce the risk of allergic conditions later in life. Cereal products, including those containing gluten, should not be administered to an infant’s diet too early (i.e. before the fourth month of life), or too late (i.e., after the seventh month of life). Some of the literature data suggest that in order to reduce the risk of developing celiac illness, type 1 diabetes, and wheat allergy, it is best to introduce these products during breastfeeding [26,27].

Meanwhile, the European Society for Paediatric Gastroenterology Hepatology and Nutrition (ESPGHAN) recommends that the extension of the child’s diet with complementary products (i.e., solids and liquids other than mother’s milk and infant formulas and follow-on formulas) should not be done before 17 weeks, but they should be introduced not later than 26 weeks [28].

As can be seen from the data available in the literature, there is no clear connection between the time of introducing solid products to the child’s diet and the occurrence of overweight or obesity in later years. Some researchers confirm positive correlations between the earlier introduction of complementary products and the increased risk of excessive weight gain leading to overweight or obesity [29,30]. However, according to most authors, there is no firm evidence that the introduction of solid products before the sixth month of life has a significant impact on the risk of overweight and obesity in childhood. Research indicates that the early introduction of complementary products to a child’s diet should be promoted for health reasons, rather than simply to satisfy the hunger of the child. In addition, the quality and quantity of food introduced should be taken into account [31,32,33].

In our own research, no relationship was found between the time of extension of the child’s diet and the prevalence of overweight or obesity in preschool age. The results indicate that in obese children the diet was extended the latest, but these were not statistically significant results, and therefore cannot be the basis for unequivocal conclusions.

In order to protect children against excessive weight gain, it is recommended that they eat products with a lower fat content. Reducing fat intake and increasing protein intake was a consequence of replacing whole milk with low-fat milk. The main reason for using low-fat dairy products is the belief that they are healthier for babies. The impact of this choice is very important because dairy products are the main source of energy for young children [34]. A different trend is recommended by Beck et al., who showed that higher consumption of milk fat is associated with a lower likelihood of severe obesity among Latino preschoolers. These results call into question the recommendations promoting the consumption of lower-fat milk [8]. Our studies confirmed the influence of the time of introducing cow’s milk into the diet on the BMI percentile in children of preschool age. Children who had cow’s milk introduced to their diets later had a lower BMI percentile.

An important issue in the development of proper eating habits in children is the start of nutrition from the so-called “big table” (or “family table”) no earlier than after 12 months of age. Meals for adults are often prepared from processed foods that contain many additives adversely affecting the development of young children, such as salt, sugar, and hot spices. Our own research showed that children who started nutrition from the family table after 12 months of life were less prone to the occurrence of overweight and obesity.

The research carried out has shown that nutritional factors in early childhood to a greater or lesser extent affect the prevalence of overweight and obesity in preschool children. In the long term, this knowledge may translate into public awareness that the food we choose for the youngest should be carefully selected and of the highest quality, because this can lead to a reduction in the risk of occurrence of a number of chronic conditions, such as overweight, obesity, and metabolic illnesses, in later years.

## 5. Conclusions

Our results suggest that extending the breastfeeding period beyond 6 months, starting to feed the child with the same meals as the rest of the family after 12 months of age, and later introduction of cow’s milk to the diet reduces the risk of the occurrence of excessive body weight in preschool children.

### Limitations

The limitation of this study is the small sample size. In future studies, it would be worth enlarging the number of examined children, especially for younger children, to have results from whole age range of preschoolers.

## Figures and Tables

**Table 1:** Characteristics of the study group.

Variables and Their Indicators	Child *N* (%)	Mother *N* (%)	Father *N* (%)
**Sex**			
Girls	162 (54.0%)	-	-
Boys	138 (46.0%)	-	-
**Age**			
4 years	30 (10.0%)	-	-
5 years	120 (40.0%)	-	-
6 years	150 (50.0%)	-	-
**Place of residence**			
Country/Village	218 (72.7%)		
Town/city	82 (27.3%)		
**Body weight category**			
Normal	230 (76.7%)	206 (68.7%)	88 (29.3%)
Overweight	26 (8.7%)	72 (24.0%)	174 (58.0%)
Obese	10 (3.3%)	22 (7.3%)	38 (12.7%)
Underweight	34 (11.3%)	0 (0.0%)	0 (0.0%)
**Education**			
Vocational	-	14 (4.7%)	34 (11.3%)
Secondary	-	126 (42.0%)	152 (50.7%)
Higher	-	160 (53.0%)	114 (38.0%)
**Infant birth size**			
AGA	256 (85.3%)	-	-
SGA	40 (13.3%)	-	-
LGA	4 (1.3%)	-	-
**Eating from the “adult table”**			
<12 months	64 (21.3%)	-	-
>12 months	236 (78.7%)	-	-
**Duration of breastfeeding**			
<6 months	188 (62.7%)	-	-
>6 months	112 (37.3%)	-	-

*N*: number of observations. Source: Own study.

**Table 2:** Relationship between body weight category and the time of introducing nutritional products and duration of breastfeeding.

Variables (Months)	Normal		Overweight and Obese		Underweight		H	*p*
	$\bar{x}$	SD	$\bar{x}$	SD	$\bar{x}$	SD		
**The time of starting to eat from the “adult table”**	14.09	5.73	13.44	5.96	15.31	5.48	0.12	0.939
**Duration of breastfeeding**	6.20	5.57	7.22	7.25	8.77	7.88	1.58	0.453
**The time of starting to eat cereals**	8.09	3.64	9.94	2.46	6.46	2.30	1.05	0.591
**The time of starting to eat vegetables**	5.43	2.13	5.72	1.64	5.46	1.27	2.36	0.306
**The time of starting to eat fruit**	5.40	2.06	5.76	1.86	4.62	1.76	0.63	0.727
**The time of starting to eat meat**	6.91	2.51	7.61	2.55	6.85	2.19	1.42	0.491
**The time of starting to drink cow’s milk**	13.70	7.04	12.06	5.71	12.62	6.54	1.62	0.444

H: ANOVA Kruskal–Wallis; *p*: level of significance of differences; SD: standard deviation; $\bar{x}$: average.

**Table 3:** Correlation of the time of introducing individual food products and duration of breastfeeding with current weight.

Variables	Current Body Weight		Current BMI Percentile	
	R	*p*	R	*p*
**The time of starting to eat from the “adult table”**	−0.01	0.877	−0.02	0.770
**Duration of breastfeeding**	−0.26	0.001	−0.15	0.064
**The time of starting to eat cereals**	0.15	0.066	0.13	0.118
**The time of starting to eat vegetables**	0.06	0.475	0.09	0.267
**The time of starting to eat fruit**	0.05	0.583	0.04	0.593
**The time of starting to eat meat**	0.05	0.546	0.07	0.425
**The time of starting to drink cow’s milk**	−0.11	0.182	−0.19	0.023

R: value of Spearman’s rank correlation.

**Table 4:** Assessment of the largest and the smallest impact of the time of introducing individual products and duration of breastfeeding on the children’s BMI percentile.

Variables	B *	R	*p*
**The time of starting to eat from the “adult table”**	−0.00	−0.00	0.959
**Duration of breastfeeding**	−0.16	−0.15	0.070
**The time of starting to eat cereals**	−0.05	−0.05	0.582
**The time of starting to eat vegetables**	0.07	0.04	0.649
**The time of starting to eat fruit**	−0.14	−0.07	0.397
**The time of starting to eat meat**	0.10	0.08	0.368
**The time of starting to drink cow’s milk**	−0.11	−0.11	0.192

b *: regression value.

**Table 5:** Odds ratio (OR) of the occurrence of overweight/obesity depending on the length of breastfeeding and eating from the “adult table”.

Parameters	The Prevalence of Overweight or Obesity	
	OR (95%CI)	*p*
**Breastfeeding**		
>6 months	1.81 (0.67–4.87)	0.003
<6 months		
**Eating from the “adult table”**		
>12 months	0.67 (0.22–2.04)	0.048
<12 months		

CI: confidence interval.

## References

[1] World Health Organization (WHO) (2010). Childhood Overweight and Obesity on the Rise.

[2] Łuszczki E., Dereń K., Baran J., Weres A., Mazur A. (2015). Trend sekularny występowania nadwagi i otyłości wśród dzieci w przedszkolach regionu rzeszowskiego. Endokrynol. Pediatryczna.

[3] Rylatt L., Cartwright T. (2016). Parental feeding behaviour and motivations regarding pre-school age children: A thematic synthesis of qualitative studies. Appetite.

[4] Yan J., Liu L., Zhu Y., Huang G., Wang P.P. (2014). The association between breastfeeding and childhood obesity: A meta-analysis. BMC Public Health.

[5] Mayer-Davis E.J., Rifas-Shiman S.L., Zhou L., Hu F.B., Colditz G.A., Gillman M.W. (2006). Breast-feeding and risk for childhood obesity: Does maternal diabetes or obesity status matter?. Diabetes Care.

[6] Arenz S., Rückerl R., Koletzko B., von Kries R. (2004). Breast-feeding and childhood obesity—A systematic review. Int. J. Obes. Relat. Metab. Disord..

[7] Stolzer J.M. (2011). Breastfeeding and obesity: A meta-analysis. Open J. Prev. Med..

[8] Beck A.L., Heyman M., Chao C., Wojcicki J. (2017). Full fat milk consumption protects against severe childhood obesity in Latinos. Prev. Med. Rep..

[9] Scharf R.J., Demmer R.T., De Boer M.D. (2013). Longitudinal evaluation of milk type consumed and weight status in preschoolers. Arch. Dis. Child..

[10] Spence L.A., Cifelli C.J., Miller G.D. (2011). The Role of Dairy Products in Healthy Weight and Body Composition in Children and Adolescents. Curr. Nutr. Food Sci..

[11] Rolls B.J., Ello-Martin J.A., Tohill B.C. (2004). What can intervention studies tell us about the relationship between fruit and vegetable consumption and weight management?. Nutr. Rev..

[12] Cooke L.J., Wardle J., Gibson E.L., Sapochnik M., Sheiham A., Lawson M. (2004). Demographic, familial and trait predictors of fruit and vegetable consumption by pre-school children. Public Health Nutr..

[13] Grimm K.A., Kim S.A., Yaroch A.L., Scanlon K.S. (2014). Fruit and vegetable intake during infancy and early childhood. Pediatrics.

[14] Okubo H., Miyake Y., Sasaki S., Tanaka K., Hirota Y. (2016). Feeding practices in early life and later intake of fruit and vegetables among Japanese toddlers: The Osaka Maternal and Child Health Study. Public Health Nutr..

[15] Möller L.M., de Hoog M.L., van Eijsden M., Gemke R.J., Vrijkotte T.G. (2013). Infant nutrition in relation to eating behaviour and fruit and vegetable intake at age 5 years. Br. J. Nutr..

[16] Kułaga Z., Różdżyńska-Świątkowska A., Grajda A., Gurzkowska B., Wojtyło M., Góźdź M., Świąder-Leśniak A., Litwin M. (2015). Percentile charts for growth and nutritional status assessment in Polish children and adolescents from birth to 18 year of age. Stand. Med. Pediatr..

[17] Jodkowska M., Woynarowska B., Oblacińska A. (2007). Test Przesiewowy Do Wykrywania Zaburzeń W Rozwoju Fizycznym U Dzieci I Młodzieży W Wieku Szkolnym.

[18] Niklasson A., Albertsson-Wikland R. (2008). Continuous growth reference from 24th week of gestation to 24 months by gender. BMC Pediatrics.

[19] World Health Organization (2002). Infant and Young Child Nutrition Global Strategy on Infant and Young Child Feeding.

[20] Papoutsou S., Savva S.C., Hunsberger M., Jilani H., Michels N., Ahrens W., Tornaritis M., Veidebaum T., Molnár D., Siani A. (2018). IDEFICS consortium. Timing of solid food introduction and association with later childhood overweight and obesity: The IDEFICS study. Matern. Child Nutr..

[21] Layte R., Bennett A., McCrory C., Kearney J. (2014). Social class variation in the predictors of rapid growth in infancy and obesity at age 3 years. Int. J. Obes. (Lond.).

[22] Grummer-Strawn L.M., Mei Z. (2004). Centers for Disease Control and Prevention Pediatric Nutrition Surveillance System. Does breastfeeding protect against pediatric overweight? Analysis of longitudinal data from the Centers for Disease Control and Prevention Pediatric Nutrition Surveillance System. Pediatrics.

[23] American Academy of Pediatrics (2012). Breastfeeding and the Use of Human Milk. Pediatrics.

[24] Gibbs B.G., Forste R. (2014). Socioeconomic status, infant feeding practices and early childhood obesity. Pediatr. Obes..

[25] Jimenez-Cruz A., Bacardi-Gascon M., Pichardo-Osuna A., Mandujano-Trujillo Z., Castillo-Ruiz O. (2010). Infant and toddlers’ feeding practices and obesity amongst low-income families in Mexico. Asia Pac. J. Clin. Nutr..

[26] Weker H., Barańska M. (2014). Żywienie Niemowląt I Małych Dzieci.

[27] Shreffler W.G., Radano M. (2011). Food allergy and complementary feeding. Nestle Nutr. Workshop Ser. Pediatric Programme.

[28] Agostoni C., Decsi T., Fewtrell M., Goulet O., Kolacek S., Koletzko B., Michaelsen K.F., Moreno L., Puntis J., Rigo J. (2008). ESPGHAN Committee on Nutrition. Complementary feeding: A commentary by the ESPGHAN Committee on Nutrition. J. Pediatric Gastroenterol. Nutr..

[29] Baker J.L., Michaelsen K.F., Rasmussen K.M., Sørensen T.I. (2004). Maternal prepregnant body mass index, duration of breastfeeding, and timing of complementary food introduction are associated with infant weight gain. Am. J. Clin. Nutr..

[30] Wilson A.C., Forsyth J.S., Greene S.A., Irvine L., Hau C., Howie P.W. (1998). Relation of infant diet to childhood health: Seven year follow up of cohort of children in Dundee infant feeding study. BMJ.

[31] Grote V., Theurich M., Koletzko B. (2012). Do complementary feeding practices predict the later risk of obesity?. Curr. Opin. Clin. Nutr. Metab. Care.

[32] Vehapoglu A., Yazıcı M., Demir A.D., Turkmen S., Nursoy M., Ozkaya E. (2014). Early infant feeding practice and childhood obesity: The relation of breast-feeding and timing of solid food introduction with childhood obesity. J. Pediatric Endocrinol. Metab..

[33] Symon B., Crichton G.E., Muhlhausler B. (2017). Does the early introduction of solids promote obesity?. Singap. Med. J..

[34] Rolland-Cachera M.F., Akrout M., Péneau S. (2016). Nutrient Intakes in Early Life and Risk of Obesity. Int. J. Environ. Res. Public Health.

